# The Relationship between the Aqueous VEGF Level and the Severity of Type 1 Retinopathy of Prematurity

**DOI:** 10.3390/jcm11185361

**Published:** 2022-09-13

**Authors:** Tianwei Liang, Zhuyun Qian, Yong Tao, Yaguang Peng, Yanhui Cui, Chengyue Zhang, Chunxia Peng, Lili Liu, Man Hu, Li Li, Ningdong Li

**Affiliations:** 1 Department of Ophthalmology, Beijing Children’s Hospital, Capital Medical University, National Center for Children’s Health, Beijing 100045, China; 2 Key Laboratory of Major Diseases in Children, Ministry of Education, Beijing 100045, China; 3 Beijing GIANTMED Medical Diagnostics Lab, Beijing 101300, China; 4 Department of Ophthalmology, Beijing Chaoyang Hospital, Capital Medical University, Beijing 100027, China; 5 Department of Clinical Epidemiology and Evidence-Based Medicine, Beijing Children’s Hospital, Capital Medical University, National Center for Children’s Health, Beijing 100045, China; 6 Department of Ophthalmology, Baoding Children’s Hospital, Baoding 071000, China

**Keywords:** retinopathy of prematurity, vascular endothelial growth factor, fundus examination, plus disease

## Abstract

Purpose: To analyze the relationship between the severity of type 1 retinopathy of prematurity (ROP) and the level of vascular endothelial growth factor (VEGF) in aqueous fluid. Methods: The aqueous VEGF levels of 49 patients (88 eyes) with type 1 ROP were retrospectively analyzed. These eyes were categorized into three groups according to the severity of disease: aggressive retinopathy of prematurity (A-ROP), threshold of ROP (T-ROP), and type 1 pre-threshold ROP (P-T-1). The differences in aqueous VEGF levels among these three groups were compared. The relationship between the aqueous VEGF level and the retinal changes of ROP, including the vessel tortuosity in zone I, and the location and stage of the ROP lesions, were also analyzed. Results: The aqueous VEGF level of the A-ROP group was the highest among the three groups, followed by those of the T-ROP and P-T-1 groups. The aqueous VEGF level was negatively correlated with the zone and the stage of the ROP diseases, while it was positively correlated with the venous tortuosity in zone I and had no relevance with the artery tortuosity in zone I. Conclusions: The aqueous VEGF level in A-ROP was the highest in type I ROP. The location of the ROP lesions and the venous tortuosity in zone I correlated with the aqueous VEGF level and could indicate the severity of ROP.

## 1. Introduction

Retinopathy of prematurity (ROP) is a retinovascular disease characterized by retinal ischemia, aberrant angiogenesis, fibrovascular proliferation, and progressive vitreoretinal traction [1]. It is a major cause of blindness in premature-birth infants. To describe the fundus change of ROP in detail and to optimize management, three concentric zones are used to describe the location of the disease, including zone I (a circle with a radius that extends from the optic disc to twice the distance from the center of the optic disc to the center of the macula), zone II (the area from the edge of zone I to the nasalora serrata), and zone III (the residual crescent of the retina anterior to zone II). In addition, five stages are graded to describe the severity of disease, including stage 1 (demarcation line), stage 2 (ridge), stage 3 (extraretinal fibrovascular proliferation), stage 4 (partial retinal detachment), and stage 5 (total retinal detachment). Lastly, plus disease is used to describe the increased venous dilation and arteriolar tortuosity of the posterior retinal vessels [2].

Type I ROP is characterized as any stage of ROP with plus disease (+), stage 3 ROP in zone I, or stages 2 or 3 ROP + in zone II [3]. It was proven that early treatment should be given to these kinds of ROP to avoid an unfavorable outcome. Until now, several studies have proven that anti-vascular endothelial growth factor (VEGF) therapy could induce the regression of ROP lesions and promote the normal angiogenesis in type I ROP [4,5,6]. Based on these results, we hypothesized that the intraocular VEGF level might be associated with the severity of type 1 ROP. To test our hypothesis, we measured the VEGF level in the aqueous humor collected from infants with type I ROP and analyzed the relationship between the aqueous VEGF level and the severity of ROP.

## 2. Methods

This retrospective case series involved infants who were diagnosed with type I ROP in Beijing Children’s Hospital (affiliated with Capital Medical University) within the period from February 2018 to February 2021. All of the infants received intravitreal anti-VEGF therapy (ranibizumab, 0.025–0.05 mL/0.25–0.5 g) (Lucentis^®^, Novartis Ophthalmics, Basel, Switzerland) as the first-line treatment. Infants who had other treatments (e.g., laser photocoagulation, cryotherapy) before anti-VEGF treatment were excluded from the study. The general characteristics of the infants, such as the gestational age (GA), birth weight (BW), and the infant’s postmenstrual age (PMA) at the time of examination, were recorded. The study was conducted in accordance with the Declaration of Helsinki. Institutional review board approval was obtained.

The involved infants underwent an ROP screening examination according to the procedure described in the previous study [7]. The ocular findings, such as the location of vascularization, the stage of acute disease, and the plus disease spectrum, were recorded in accordance with the International Classification of ROP, Third Edition (ICROP3) [8]. The artery tortuosity in zone I was graded as mild (tortuosity angle > 90°), moderate (tortuosity angle = 90°), and severe (tortuosity angle < 90°). The venous tortuosity in zone I was also graded based on the same criteria. If more than one grade of vessel tortuosity was present in the same eye, only the most severe grade was taken into analysis. The severity of ROP was categorized into three groups: aggressive retinopathy of prematurity (A-ROP; posterior location of retinopathy and prominence of plus disease), threshold ROP (T-ROP; at least 5 continuous or 8 cumulative hours of stage 3 ROP in zone 1 or 2, with plus disease), and type 1 pre-threshold ROP (P-T-1; zone 1 any-stage ROP with plus disease, zone 1 stage 3 ROP with or without plus disease, and zone 2 stage 2 or 3 ROP with plus disease) [3,8].

The aqueous humor was obtained before the intravitreal injection of an anti-VEGF agent. About 50–100 μL of aqueous humor was obtained through paracentesis using a 29-gauge needle. The aqueous VEGF level was measured using the Becton Dickinson Cytometric Bead Array bead-based immunoassay (Human VEGF Flex Set, No. 558336, BD Bioscience, San Jose, CA), following the manufacturer’s instructions. Briefly, VEGF capture beads were incubated with calibrators (standards ranging from 0 to 2500 pg/mL) or the aqueous humor in the dark. Anti-VEGF phycoerythrin-labeled antibody was then added and the mixture was subsequently incubated in the dark for 2 h. After washing, two-color flow cytometry analysis was performed, and data were acquired and analyzed using the Becton Dickinson FCAP Array software. The VEGF concentrations in the aqueous humor from the patients and controls were determined from the standard curves.

Statistical analyses were carried out using the SPSS software for Windows (version 28.0; IBM-SPSS, Chicago, IL, USA). The normality of data was tested using the Kolmogorov–Smirnov test. Spearman’s correlation coefficient was used to analyze the correlation between the GA, BW, and PMA of included infants and the aqueous VEGF level. The partial correlation coefficient was used to analyze the correlation between the ocular findings of ROP (the location of ROP lesions, the stage of ROP lesions, and the grade of vessel tortuosity in zone I) and the aqueous VEGF level. Multiple linear regression analysis was further used to determine the relationship between the above-mentioned variables and the aqueous VEGF level. The one-way ANOVA analysis was used for the comparison of aqueous VEGF levels among different ROP groups. All of the *p*-values less than 0.05 were considered statistically significant, and all of the tests were two-sided.

## 3. Results

**Figure 1:**
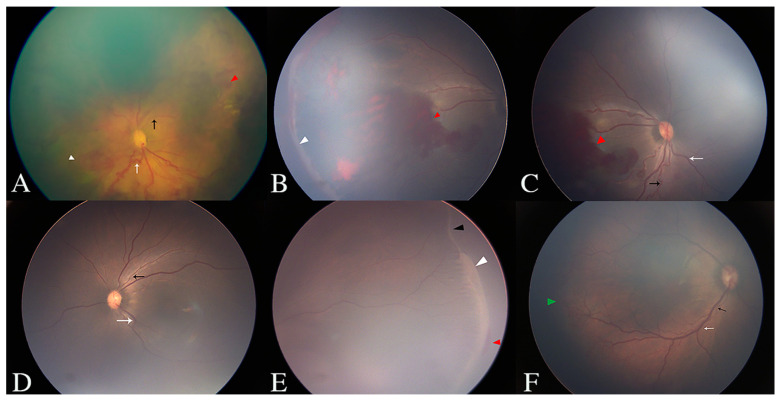
Fundus photographs demonstrating the results of the fundus examinations, including the vessel tortuosity in zone I, and the location and stage of ROP lesions. (**A**) Female, GA 33 W + 6, BW 1900 g, PMA 38 W, right eye, A-ROP with zone I stage 3 disease (white arrowhead) and intraretinal hemorrhage (red arrowhead). Artery and venous tortuosity were graded as mild (black arrow) and severe (white arrow), respectively. (**B**,**C**) Male, GA 30 W + 4, BW 1500 g, PMA 41 W + 2, right eye, T-ROP with anterior zone II stage 3 disease (white arrowhead) and intraretinal hemorrhage (red arrowhead). Artery and venous tortuosity were graded as severe (black arrow) and mild (white arrow), respectively. (**D**,**E**) Male, GA 30 W + 4, BW 1500 g, PMA 41 W + 2, left eye, P-T-1 with anterior zone II stage 3 disease (white arrowhead) and intraretinal hemorrhage (red arrowhead). Artery and venous tortuosity were graded as mild (black arrow) and moderate (white arrow), respectively. Stage 2 disease (black arrowhead) was also presented in this eye. (**F**) Male, GA 30 W + 5, BW 1700 g, PMA 36 W + 6, right eye, P-T-1 with posterior zone II stage 1 disease (green arrowhead). Artery (black arrow) and venous (white arrow) tortuosity were both graded as moderate.

Forty-nine infants (88 eyes) were included in this study. The GA ranged from 25.7 to 33.9 weeks, with a mean GA of 29.1 ± 1.8 weeks and a median GA of 29 weeks. The BW ranged from 760 to 2800 g, with a mean BW of 1300 ± 376 g and a median BW of 1255 g. The PMA ranged from 33.1 to 47 weeks, with a mean PMA of 39.9 ± 3.3 weeks and a median PMA of 40.1 weeks. The mean aqueous VEGF level of all involved eyes was 278.2 ± 492.1 pg/mL (range: 1.3–3824.1 pg/mL), with a median value of 128.9 pg/mL. Detailed results of the fundus examinations, including the vessel tortuosity in zone I, and the location and stage of the ROP lesions, are listed in Table 1 and shown in Figure 1. Data of the corresponding aqueous VEGF levels are also listed in Table 1. 

**Figure 2:**
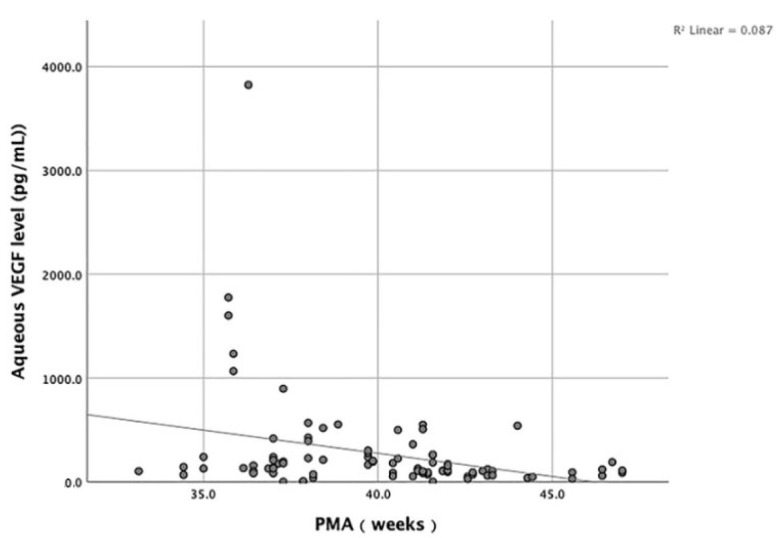
Scatterplot showing the negative correlation between the PMA and the aqueous VEGF level.

We analyzed the correlation between the GA, BW, and PMA of included infants and the aqueous VEGF level. The results show that no significant correlation existed between the GA, BW, and the aqueous VEGF level (*p* > 0.05). On the contrary, the PMA was negatively correlated with the aqueous VEGF level (*p* = 0.001, R= −0.346) (Figure 2).

According to the severity of ROP, 17 eyes, 11 eyes, and 60 eyes were categorized as A-ROP, T-ROP, and P-T-1, respectively. The mean aqueous VEGF level was 747.5 ± 976.1 pg/mL in the A-ROP group, 268.0 ± 199.0 pg/mL in the T-ROP group, and 147.1 ± 105.3 pg/mL in the P-T-1 group, with a median value of 425.8 pg/mL, 212.0 pg/mL, and 110.0 pg/mL, respectively. Significant differences were found among the three groups (*p* = 0.000). Further pairwise comparison showed that the aqueous VEGF level in the A-ROP group was significantly higher than those in the T-ROP group (*p* = 0.006) and in the P-T-1 group (*p* = 0.000). However, no significant difference in aqueous VEGF level was found between the T-ROP and P-T-1 groups (*p* = 0.402).

The zone, stage and the plus disease spectrum were important parameters to evaluate the severity of ROP and could have relevance to the aqueous VEGF level. To avoid potential bias, we used the partial correlation coefficient to analyze the relationship between these parameters and the aqueous VEGF level. The results show that the aqueous VEGF level was negatively correlated with the zone of ROP lesions (*p* = 0.000, r = −0.480) as well as with the stage of ROP lesions (*p* = 0.013, r = −0.268). The aqueous VEGF level was positively correlated with the venous tortuosity in zone I (*p* = 0.015 r = 0.263) and had no relevance with the artery tortuosity in zone I (*p* = 0.178).

According to the multiple linear regression analysis, the zone of ROP lesions, the stage of ROP lesions, and the grade of venous tortuosity in zone I significantly affected the aqueous VEGF level. The variable that most positively affected the aqueous VEGF level was the grade of venous tortuosity in zone I. On the contrary, the variable that most negatively affected the aqueous VEGF level was the zone of ROP lesions. The multiple linear regression analysis results of the variables affecting the aqueous VEGF level are presented in Table 2.

## 4. Discussion

The pathogenesis of ROP was divided into two phases. In phase I, the delayed physiological retinal vascular development results in a peripheral avascular area, and in phase II, intravitreal angiogenesis occurs at the junction of the avascularized and vascularized retinas [9]. Vascular endothelial growth factor (VEGF) is the principal mediator of pathological angiogenesis [10]. It is downregulated in phase I when a premature-birth infant is exposed to a relatively high-oxygen environment after birth, and is upregulated by an avascularized retina in phase II [11]. Based on these theories, we measured the aqueous VEGF level in type 1 ROP and analyzed the relationship between the aqueous VEGF level and the severity of retinopathy of prematurity.

The GA and BW are two major risk factors for the occurrence of ROP [12]. In the CRYO-ROP cohort, each 100 g increase in BW decreased the odds of reaching the threshold ROP by 27%, and each weekly increase in GA decreased the odds of reaching the threshold disease by 19% [13]. In our study, the GA and BW did not correlate with the aqueous VEGF level. One possible explanation for this was that the aqueous humor sample was not collected soon enough after infant birth. Medical interventions after birth might have added bias to the correlation analysis. Another possible explanation was that the GA and BW influenced the development and progression of ROP via other physiopathologic mechanisms. For example, several inflammatory parameters in plasma, including interleukin (IL)-8, monocyte chemoattractant protein (MCP)-1, alkaline phosphatase (AP), and IL-1β, were proven to be correlated with GA and BW in preterm infants [14]. The role of inflammation in the development of ROP was also proven in both the plasma and the intraocular fluid [15,16].

In our study, the aqueous VEGF level was the highest in the A-ROP group, followed by those of the T-ROP and P-T-1 groups, which could explain the most aggressive retinopathy in A-ROP. A-ROP is the most severe form of type 1 ROP, and it can rapidly progress to tractional retinal detachment (RD). Nedime et al. [17], in a study involving 15 eyes with A-ROP, administered intravitreal injections of ranibizumab (0.25 mg/0.025 mL). All eyes developed plus disease and received retreatment during the follow-up visit. Tong et al. [18] reported a case series of 160 eyes diagnosed with A-ROP and that had received an intravitreal injection of ranibizumab (0.3 mg/0.03 mL). The RD occurrence rate was 21.6%, and the rate of multiple intravitreal injections was 65.5%. Based on our finding that the aqueous VEGF level in the A-ROP group was much higher than those in the T-ROP and P-T-1 groups, we propose that the conventional dose of intravitreal anti-VEGF agent might be insufficient to neutralize the intraocular VEGF in eyes with A-ROP, and thus cause the rate of reactivation to remain high even after administering timely anti-VEGF treatment. Since the neurodevelopmental outcomes in preterm infants after intravitreal injection of anti-VEGF agents remains unclear [19,20,21,22], the optimal dose of intravitreal anti-VEGF agent for A-ROP infants still needs to be investigated in future studies.

Our study shows that the aqueous VEGF level in the T-ROP group was higher than in the P-T-1 group, although no significant difference was found. This result is consistent with the study of Lyu et al. [16] in 2018. The results of the CRYO-ROP study show an approximate 50% risk of retinal detachment in T-ROP if not treated [23]. In the ETROP study, early treatment of pre-threshold type 1 ROP was also recommended for a better visual prognosis and retinal structure [3]. Our VEGF level findings in the T-ROP and P-T-1 groups further verified the necessity of early treatment for T-ROP and P-T-1 on the molecular level.

We analyzed the relationship between the aqueous VEGF level and the fundus changes of ROP, including the morphological change in the posterior retinal vessels, and the location and stage of ROP lesions. Our results show that eyes with ROP lesions closer to the posterior region had a higher aqueous VEGF level, which proved the more severe disease of zone I ROP. It was notable that eyes with a higher stage of ROP lesion had a lower aqueous VEGF level, which did not seem to conform with the natural course of ROP. The possible reason for this was that we only involved type I ROP in this study, which meant that eyes with stage I ROP had zone I disease and the most severe plus disease, simultaneously, which caused the selection bias. We also found that it was the venous tortuosity, but not the artery tortuosity, which positively correlated with the aqueous VEGF level. In another study of central retinal vein occlusion, the aqueous VEGF concentration was also proven to be significantly correlated with the venous tortuosity [24]. We propose that retinal venous tortuosity is correlated with the degree of circulatory disorder and retinal ischemia, and could further be used as an indicator to identify eyes with more severe ROP. 

In summary, we measured the aqueous VEGF level of type 1 ROP and found that the aqueous VEGF level in A-ROP was the highest in type I ROP. The location of the ROP lesions and the venous tortuosity in zone I correlated with the aqueous VEGF level, and could indicate the severity of ROP.

Our study had some limitations. First, its retrospective design introduced a potential selection bias. Second, the dilation of the posterior retinal vessels was not analyzed due to the lack of image analysis methods used. Third, the aqueous sample was collected only before the first intravitreal injection, and the variation in the aqueous VEGF level was not investigated. In the future, we intend to conduct studies with prospective design and different doses of intravitreal anti-VEGF agent to determine the optimal dose for A-ROP. Furthermore, the concentration of VEGF and other inflammatory cytokines in the intraocular fluid sample should be measured to further explore the interaction among these cytokines and their effect on the progression of ROP.

## Figures and Tables

**Table 1:** Detailed results of the fundus examinations and the corresponding aqueous VEGF levels.

Examination Parameter	Eyes	Mean Aqueous VEGF Level (pg/mL)	Median Aqueous VEGF Level (pg/mL)
The location of ROP lesions, *n* (%)			
Zone I	11 (12.5)	1085.0 ± 1070.6	897.1
Posterior zone II	15 (17.0)	188.6 ± 181.8	138.4
Anterior zone II	62 (70.5)	156.8 ± 124.8	109.4
The stage of ROP lesions, *n* (%)			
Stage I	5 (5.7)	1500.9 ± 1510.3	1601.4
Stage II	39 (44.3)	138.6 ± 80.8	110.5
Stage III	44 (50)	263.0 ± 283.4	148.8
Artery tortuosity in zone I, *n* (%)			
Mild	41 (46.6)	177.4 ± 149.9	119.2
Moderate	17 (19.3)	197.5 ± 153.1	186.2
Severe	30 (34.1)	461.8 ± 793.4	127.7
Venous tortuosity in zone I, *n* (%)			
Mild	74 (84.1)	243.5 ± 331.4	127.9
Moderate	9 (10.2)	208.0 ± 174.7	212.0
Severe	5 (5.7)	919.6 ± 1629.3	142.8

ROP, retinopathy of prematurity; VEGF, vascular endothelial growth factor.

**Table 2:** Multiple linear regression analysis results of the variables affecting the aqueous VEGF level.

Aqueous VEGF Level	Non-Standardized Coefficients		Standardized Coefficients	T	*p*
	Beta	Std. Error	Beta		
Zone of ROP lesions	−341.936	73.101	−0.491	−4.678	0.000
Stage of ROP lesions	−212.287	74.533	−0.269	−2.848	0.006
Grade of venous tortuosity in zone I	213.189	83.645	0.232	2.549	0.013

ROP, retinopathy of prematurity; VEGF, vascular endothelial growth factor.

## Data Availability

All data generated or analyzed during this study are included in this published article.
